# Supplementary material for: Patterns in artisanal coral reef fisheries revealed through local monitoring efforts
Source: PeerJ. 2017 Dec 4;5:e4089. doi: 10.7717/peerj.4089 (PMC5719965; doi:10.7717/peerj.4089)
Supplement: Supplemental Information 1 [file peerj-05-4089-s001.docx]

## S1 Appendix: Source information for effort, catch and catch-per-unit-effort (CPUE) estimates

### Effort estimates

For effort, we produced a set of estimates for spear, line, and net gear types and, if possible given available information, these gear-specific estimates did not include nightime and vessel-based fishing activities (i.e., surround net or trolling).

For Hanalei, we estimated gear-specific shore-based fishing effort with information from Table 2 as seen in Everson and Friedlander (2004) for the period of spring 1993 to winter 1993. Line fishing only included values for pole-and-line for that year as we assumed that all other line fishing was vessel-based. Also, all nets were included in the overall shoreline net estimate except small surround net, as we assumed it was vessel-based. We further assumed all other types of nets observed were not boat-based.

For Kahekili, we used data from Table 3 as reported by Friedlander et al. (2012) to obtain effort estimates. For gear-specific estimates, we used each of the three gear types separately. The hours under the category of “other” were excluded from the line, net, and spear categories.

For Kailua and Waimānalo, we used a yearly average from more than 5 years of monitoring (K. Stamoulis pers. comm.). For the shore-based total effort, we excluded surround nets and vessel-based fishing including trolling and boat-based line fishing (labeled as “line” in the file). In the estimate for effort for non-vessel-based nets, we included all net types that included the word “net” and also seine in the dataset, except surround nets. Trammel nets were grouped with gill nets, which were included in our estimate of net fishing effort. Data from Kailua included the effort recorded for Lanikai. The average daily effort estimates for Kailua and Waimānalo were quantified for weekends/holidays and weekdays separately and then expanded by multiplying the average number of days per year of these two day types over the study period to obtain average yearly effort for these strata. We then summed these two values to obtain a total annual average estimate of fishing effort for these two windward locations (K. Stamoulis pers. comm.).

Effort estimates were produced from the intercept data from the Kaloko-Honokōhau National Historical Park adjusted to a 12-hour day of monitoring and analyzed using methods of Friedlander and Parrish (1997). The data were analyzed separately by day type (weekday, weekend/holidays) then expanded for the number of each day type in 2010. The total effort was the sum of effort from weekdays and weekend/holidays of 2010.

For Kāne‘ohe Bay, we used data from Everson and Friedlander’s Table 1 (Everson & Friedlander, 2004). For line, we did not include trolling but the estimate of “Pole–and-line”, which seems to combine shore and boat-based. For net, we used all gears that had the word “net” included in their label and were quantified in effort-hours for nets. Gill nets were observed but were not included in our estimate as they were estimated in effort-days rather than gear-hours as other gears were quantified.

For Ka‘ūpūlehu, we summed values from Table 2 produced by Koike et al. (2015). All gear types of line fishing were included in the total estimate for the non-vessel based fishing effort estimates except trolling which was excluded from line fishing (which included whipping, slide bait, dunking, bamboo, and hand pole). For net, we combined estimates of effort from throw net and scoop net. For spear, we combined the effort estimates of three-prong and spear estimates. The category of “others” was not included as line, net, and spear fishing (Koike et al., 2015).

For Kīholo, we derived the effort estimates from Table 2 as produced by Kittinger et al. (2015). For line fishing, we combined estimates from hand pole and rod and reel. Other than crabbing that was included in net fishing effort, all other fishing methods listed as “other” were not included as line, net, or spear fishing.

For Maunalua Bay, we used the effort estimates generated by K. McCoy (pers. comm.). We used only daytime effort estimates to be most comparable to other creel surveys. From the daytime data, we multiplied the average gear units by the average time that they are used and then multiplied this product by 365 for the days in a year to produce an annual estimate. For the estimate of total effort for nets, we added the daytime effort for scope and throw nets.

For Pearl Habor, we used effort estimates from Table 6 (Wolfe et al., 2017). We did not include effort from the categories of “Vessel”, “Trap”, and “Glean”.

Two creel surveys were conducted at Puakō. For the creel survey conducted in Puakō from 2008-2009, we estimated values from raw data produced by J. Giddens (pers. comm.). The estimated daily fishing effort was calculated as the product of the mean complete fishing trip duration for each gear type and the mean gear-hours observed per day. Because surveys took place over 2.5 hours, this estimate of effort per day was expanded by a factor of 4.8 to obtain the total daily fishing effort for a 12-hour period. Estimated daily effort was expanded by the number of days available in the year. Weekday and weekend/holiday effort were treated separately and summed to produce the total annual effort estimate. Then we collated the multiple gear types into the three gears types to determine the total effort in hours per year as reconstructed. All line fishing gear types were included except trolling (i.e., bamboo, hand line, pole and line, rod and reel dunk, rod and reel whip). Spear included scuba spear and spear. Net included all types of nets. This is a conservative estimate of fishing efforts.

For the Puakō survey conducted from 1980 to 1981 (Hayes et al., 1982), we obtained effort values for line and spear by taking average values from the annual estimates for the two years (1 May 1980 to 30 April 1981 and 1 September 1980 to 31 August 1981) at the top of Table 49 (Hayes et al., 1982). For our estimate of spear fishing effort from the older Puakō survey, we included spear fishing effort for both fish and octopus. We could not estimate effort for nets as gill net and throw nets as these estimates were quantified as sets and casts, respectively (Hayes et al., 1982).

For Pūpūkea, Stamoulis and Friedlander (2013) monitored the fishing effort in the summer of 2011 (June-August). Because this site experiences large waves that prevent or limit fishing activity during other seasons of the year, we used a scaled proportion of observed summer fishing effort based on the wave energy during the spring, fall, and winter months of 2011. Monthly average wave energy records from 2011 were obtained from the Pacific Islands Ocean Observing System (PacIOOS) Voyager Waimea wave buoy through the PacIOOS Voyager website (<http://oos.soest.hawaii.edu/pacioos/voyager/>). Months with the highest wave energy (November-March) were assumed to have zero fishing effort. September was attributed 75% (3/4) of summer fishing levels, while April and September were estimated to have 67% (2/3) of summer fishing effort (K. Stamoulis pers. comm.).

For Wailuku, we summed legal and illegal fishing effort for each of the gear types as recorded in Fig. 6 (Koike, Carpio & Friedlander, 2014). For line fishing, we included effort from hand pole and rod and reel. For effort from nets, we included effort from throw net, laynet, and opae net.

### Catch estimates:

For catch estimates, we obtained overall estimates of annual coral reef fisheries and invertebrate harvest. If possible given available information (i.e., reported biomass of the following species), we estimated the proportion of the total catch for bigeye scad (*Selar crumenophthalmus*) and octopus (*Octopus cyanea* and *Callistoctopus ornatus*).

For Hanalei Bay (15,801 kg), we took this value from the text in Fig. 3 produced by Everson and Friedlander (2004). The catch of *S. crumenophthalmus* was obtained from Table 5 (Everson & Friedlander, 2004). These two values allowed us to produce our estimate of catch of *S. crumenophthalmus* for Hanalei Bay.

For Kahekili, the total catch is reported in the executive summary produced by Friedlander et al. (2012). We obtained the biomass of octopus from Table 5 (Friedlander et al., 2012).

Catch estimates were produced from the intercept data from Kaloko-Honokōhau National Historical Park, adjusted to a 12-hour day of monitoring and analyzed using methods of Friedlander and Parrish (1997). The data were analyzed using a day type stratum of weekday and weekend / holidays separately and then expanded for the number of each day type in 2010 and summed to provide an annual estimate of total catch. Octopus was caught and recorded, so we could estimate the percent of total catch that was octopus in our analysis.

For Kāne‘ohe Bay, we took this value from the text in Fig. 3 produced by Everson and Friedlander (2004). Estimates for octopus and *S. crumenophthalmus* were obtained from the “Average 1991-92” column of Table 4 (Everson & Friedlander, 2004).

For Ka‘ūpūlehu, we took the total catch reported in the executive summary produced by Koike et al. (2015) and converted to it from lb to kg. We obtained the estimate of *S. crumenophthalmus* (referred to as “Akule”) and octopus (referred to as “Day tako”) from Table 4 (Koike et al., 2015), and converted from lb to kg. The value for Kīholo was taken from the abstract of Kittinger et al. (2015). Expanded catch was only reported by trophic group, so our catch estimates included catch data for total catch reported.

For Maunalua Bay, estimates were provided by K. McCoy (pers. comm.). We used biomass caught during the day and night fishing catch. These values excluded catch of *S. crumenophthalmus*, mackerel scad (*Decapterus* spp.), and octopus (*Octopus cyanea* and *Callistoctopus ornatus*) (K. McCoy, pers. comm.).

For Pearl Habor, we used the total of the year, which is in the bottom right cell of Table 8 produced by Wolfe et al. (2017). We obtained the biomass of octopus as seen in Table 9 (Wolfe et al., 2017). No *S. crumenophthalmus* was observed being caught during the survey and no catch was estimated for net, as fishers using this gear were not interviewed since a) there was a limited numbers of fishers using nets, and b) the main net fisher that was observed always entered / exited from an access point that was difficult to reach (B. Wolfe, pers. comm.).

For the Puakō survey conducted in 2008-2009, J. Giddens (pers. comm.) estimated total reconstructed catch as the product of the annual effort per gear type (as a proportion of the total effort), and the average CPUE for that gear type. Octopus were caught but not included in the estimate of catch. No *S. crumenophthalmus* was observed being caught during the survey.

For the Puakō survey conducted in 1980-1981, we converted values in the “Total” column for overall catch in Table 50 (Hayes et al., 1982) from number of individuals of each species (Hayes et al., 1982) to its biomass by multiplying the number of fish by an estimated average weight for that species. Average weights were taken from fish surveys conducted at Puakō from 2004-2011 (K. Stamoulis pers. comm.), Kittinger et al. (2015) that had average weights for fish caught in Kīholo, and estimated weights from Kaloko-Honokōhau National Historical Park creel survey. To get total catch, we summed the weights for all species and gear types. With this approach, we were able to estimate biomass of octopus that was reported.

For Waikīkī, the three values for the three sites were obtained from the “Total” row of Table 4.10 as produced by Meyer (2003). These values were in tonnes, so they were converted to estimates of catch in kg.

For Wailuku, we summed the total catch estimates for all the gear types at the bottom of Table 3 (Koike, Carpio & Friedlander, 2014) then converted the estimates from lb to kg. The estimate of the biomass of *S. crumenophthalmus* and octopus (he‘e) was obtained from Table 4 (Koike, Carpio & Friedlander, 2014), which we converted from lb to kg.

**CPUE estimates:**

We produced a set of CPUE estimates for spear, line, and net gear types and, if possible given available information, these gear-specific estimates did not include vessel-based fishing activities (i.e., surround net or trolling).

The CPUE estimates for Hā‘ena were produced in kg / hr by K. McCoy (pers. comm.). These CPUE values were only estimated for line, net, and spear and excluded data from bigeye scad (*S. crumenophthalmus*), mackerel scad (*Decapterus* spp.), and octopus (*O. cyanea* and *C. ornatus*) (K. McCoy, pers. comm.).

For Hanalei Bay, we used CPUE estimates from Table 2 of Friedlander and Parrish (1997). For line we used “lines from shore”, for nets we used “cast net”, “gill net”, and “crab net”, and for spear we used “spear”.

For Kahekili, we used the CPUE estimates from Table 4 produced by Friedlander et al. (2012).

CPUE estimates were produced from the intercept data from Kaloko-Honokōhau National Historical Park adjusted to a 12-hour day of monitoring and analyzed using methods of Friedlander and Parrish (1997). The CPUE estimates were estimated for the three gear types (line, net, and spear) as average daily CPUE estimates. These values were produced with no strata of day type (all intercept data pooled).

For Kāne‘ohe Bay, we used CPUE estimates from the “Average 91-92” column in Table 3 of Everson and Friedlander (2004). For line we used the estimate of “Line fishing”, which combines shore and boat-based fishing, but we did not include the estimate for trolling. There was no CPUE for throw net, so we used crab nets as CPUE estimate for our overall net category reported by Everson and Friedlander (2004).

For Ka‘ūpūlehu, we used the CPUE estimates and sample sizes from the figure on the 11^th^ page of the PDF produced by Koike et al. (2015). From this figure, we estimated averages for line (included data from whipping, dunking, bamboo, and slide bait), net (included data from scoop net and throw net) and spear (included data from spear gun and three-prong) and then converted from lb per hour to kg per hour.

For Kīholo, we used the single category of spear and throw net for spear and net, respectively, as seen in the yearly mean column of the S6 Table of Kittinger et al. (2015). For line, we used the CPUE estimates and sample sizes from both rod and reel and hand pole as seen in the S6 Table to estimate the CPUE for line fishing (Kittinger et al., 2015).

The CPUE estimates for Maunalua Bay were produced by K. McCoy (pers. comm.). These values were only estimated for line, net, and spear and excluded data on bigeye scad (*S. crumenophthalmus*), mackerel scad (*Decapterus* spp.), and octopus (*O. cyanea* and *C. ornatus*) (K. McCoy, pers. comm.). She produced her estimates as lb / gear-hour (K. McCoy, pers. comm.), so we converted these estimates to kg / gear-hour. For line fishing we used the estimate from “line” and for spear we used “spear”. For the overall net CPUE, we produced an average value using the sample sizes and daytime estimates for throw, lay, and scoop nets.

For Pearl Habor, the CPUE estimates of line and spear fishing were obtained from Table 7 of Wolfe et al. (2017). No CPUE values was estimated for nets as catch for this gear type was not estimated due to a lack of intercepts with net fishers during the study (Wolfe et al., 2017).

The CPUE estimates for the Puakō survey conducted from 2008-2009 were produced by J. Giddens (pers. comm). Catch-per-unit-effort (kg / gear-hour) was calculated by dividing the catch in weight by the gear-hours spent fishing. Mean CPUE for each species and each gear-type was estimated by summing CPUE over all interviews and dividing by the number of interviews. Octopus were caught but not included in the CPUE estimates. For the 1980-1981 Puakō creel survey, we divided the total catch of each gear approach by the total effort for that gear approach. We describe how those values were derived as already noted by converting values from Tables 49 and 50 (Hayes et al., 1982) to annual effort and catch that we used. No CPUE estimates were produced for nets as estimates of effort were quantified in casts or sets, rather than hours (Hayes et al., 1982).

For Waikīkī, the CPUE estimates for line and spear were reported in Table 4.11 produced by Meyer (2003) but no value was reported for net, so we left it blank. The table confirms that the CPUE for line fishing was shore-based only (Meyer, 2003).

For Wailuku, we took CPUE estimates from Fig. 4 (Koike, Carpio & Friedlander, 2014) and converted to kg / gear-hours. Given that there was no cast net / throw net reported and only one net CPUE was reported, we used opae net for the CPUE of net. For line fishing, Fig. 4 (Koike, Carpio & Friedlander, 2014) documents CPUE estimates and sample sizes for both rod and reel and hand line, so we averaged these values.

### Fish flow estimates:

For Hā‘ena, from the unpublished report produced by Kuaʻāina Ulu ʻAuamo (KUA), Hui Makaʻāinana o Makana and Limahuli Gardens staff (unpublished report), we used the data in Table 12 by dividing the frequency of each category by the total sample size of all categories combined. “Mahele” was used as given away for home consumption. For Hanalei, we used values from the “Mean” row of overall disposition of catch among interviewees reported in the bottom row of Table 4-6 (Glazier & Kittinger, 2012).

For Kāne‘ohe Bay, we used data from all gear types listed in Table 6 as produced by Everson and Friedlander (2004). For values reported as “<1”%, we used a value of 0.5% in producing our estimates of mean disposition of catch. For Kāne‘ohe Bay, the category of “% other” indicated that part of the catch was either given away or used as bait. With these data, we produced a set of mean values.

For Kīholo, these values were obtained from the abstract of Kittinger et al. (2015). For Maunalua Bay, these values were obtained from Table 5 that was produced by Kittinger (2013).

For Puakō, we summarized data from Table 7 produced by Giddens (2010). The proportion of catch was produced by dividing the frequency of response by total sample size. Our category of “Other” included the categories of “Unknown”, “Home Aquarium”, and “Test for Ciguatera” (Giddens, 2010).

Fish flow information for additional areas of Hawai‘i Island were obtained from Fig. 18 as produced by Hardt (2011).

For Wailuku, this information came from the Executive Summary of the report produced by Koike et al. (2014). Given that only two categories were reported, these added up to only 99%. Therefore, we assumed this was due to rounding and to correct for this, we added 0.5% to each of the the two values that were reported.
